# Vibronic and Cation Spectra of Cyclopropylbenzene Conformer

**DOI:** 10.3390/molecules31101658

**Published:** 2026-05-14

**Authors:** Zefeng Hua, Xiaokang Ma, Zhixie Wang, Yiwen Xie, Kunwu Shen, Jing Zhou, Zhongfa Sun, Xinyan Yang, Zhengbo Qin, Xianfeng Zheng

**Affiliations:** Anhui Province Key Laboratory for Control and Applications of Optoelectronic Information Materials, School of Physics and Electronic Information, Anhui Normal University, Wuhu 241002, China; xiaokma2026@163.com (X.M.); wangzhixie@ahnu.edu.cn (Z.W.); wszlhd@ahnu.edu.cn (Y.X.); 2521011199@ahnu.edu.cn (K.S.); zhoujing030305@163.com (J.Z.); zfsun@ahnu.edu.cn (Z.S.); xinyanyang@ahnu.edu.cn (X.Y.)

**Keywords:** multiphoton ionization, photoelectron imaging, vibrational assignment, cyclopropylbenzene (CPB)

## Abstract

The vibronic spectra of the first excited singlet state (S_1_) and the cation spectra of the ground state of the cation (D_0_) of jet-cooled cyclopropylbenzene (CPB) were investigated using resonance-enhanced multiphoton ionization (REMPI) and photoelectron velocity-map imaging techniques, respectively. The vibronic spectra indicated the existence of only the bisected conformer, a finding corroborated by quantum chemical calculations. The S_0_ → S_1_ electronic transition originated at 36,858.5 cm^−1^, with an adiabatic ionization energy of 66,846 ± 15 cm^−1^. Vibrational levels in both states were assigned with the assistance of theoretical geometry optimization and frequency calculations. These experimental spectra and theoretical calculations provided valuable insights into the structural and vibrational characteristics of CPB in its excited and cationic states.

## 1. Introduction

Cyclopropylbenzene (CPB), represented by the chemical formula C_6_H_5_-C_3_H_5_, consists of a benzene ring bonded to a cyclopropyl group. Due to their unique high ring strain of the three-membered structures, cyclopropyl rings are widely employed as versatile building blocks in the synthesis of numerous pharmaceutical compounds. Cyclopropane-containing molecules are of significant importance in the development of pharmaceutical products [1,2]. Moreover, natural products containing cyclopropyl rings have been found to possess a broader range of biological activities [3,4]. Beyond synthetic applications, the electronic structure of the cyclopropyl group itself has been a subject of fundamental interest. Recent experimental and theoretical studies have provided compelling evidence that the cyclopropyl cation possesses a transition-state nature via its disrotatory ring-opening pathway [5], highlighting the unique electronic characteristics that distinguish cyclopropane from simple alkyl groups.

The conformational preference of CPB is governed by the orbital interaction between the cyclopropyl HOMO (highest occupied molecular orbital, Walsh orbital) and the phenyl π system. In the bisected conformation, maximal overlap between the donor cyclopropyl Walsh orbital and the acceptor phenyl lowest unoccupied molecular orbital (LUMO) is achieved, lowering the HOMO-LUMO gap and promoting charge transfer in the excited state. The perpendicular conformation, by contrast, minimizes this interaction. This conformational sensitivity has been demonstrated in related systems: studies on *para*-cyclopropyl-substituted phenyltropylium ions revealed that the bisected arrangement provides substantially greater cation stabilization than the perpendicular form, as evidenced by ^1^H NMR spectroscopy and p*K*_R_^+^ measurements [6]. Such conformational effects on orbital interactions make CPB an ideal model system for studying cyclopropyl-aryl conjugation.

Previous spectroscopic and theoretical studies have explored the CPB molecular energy levels and determine its conformation. Fourier transform microwave spectra were utilized to record the rotational spectra of jet-cooled CPB, revealing a bisected conformer, with no evidence of a perpendicular conformer [7]. However, theoretical calculations have suggested the existence of both bisected and perpendicular forms. In the first systematic search for CPB conformers via electronic spectroscopy, Philis and Wategaonkar recorded the S_0_ → S_1_ transition using both (1 + 1) and (2 + 2) REMPI techniques and detected only the bisected conformer [8]. More recently, Villani and colleagues reinvestigated CPB using a comprehensive multi-technique approach combining 1c-R2PI, UV-UV hole-burning, and IR-UV ion depletion spectroscopy in conjunction with quantum chemical calculations [9]. Their analysis of the cyclopropyl CH stretch region, aided by a local anharmonic model based on density functional theory calculations, provided definitive assignment to the bisected conformer. The absence of the perpendicular conformer was attributed to low computed barriers to conformer interconversion [9]. Conformational studies on related substituted benzenes—sec-butylbenzene [10], phenylpropyne [11], and p-cymene [12]—have employed similar jet-cooled spectroscopic approaches. However, CPB is distinguished from these systems by the pronounced Walsh orbital character of the cyclopropyl substituent, which introduces a degree of electronic coupling absent in typical alkylbenzenens.

Despite these advances in characterizing the neutral CPB molecule, spectroscopic investigations in its cationic state remain notably scarce. The ionization process removes an electron from the HOMO, which in the bisected conformer has significant cyclopropyl Walsh orbital character, potentially leading to substantial geometry changes upon ionization. Knowledge of the cation ground state (D_0_) vibrational structure is essential for understanding the charge redistribution upon ionization and the stability of the bisected conformation in the ionic manifold. Furthermore, precise determination of the adiabatic ionization energy, which constrains the thermochemistry of CPB-derived radical cations relevant to mass spectroscopic fragmentation pathways, as demonstrated for related cyclopropyl-containing cations [13], now enable accurate prediction of spectroscopic observables to guide and validate experimental assignments.

In this study, we performed theoretical calculations to investigate the molecular structures of the CPB molecule in the S_0_, S_1_, and D_0_ states. The optimization process provided detailed insights into the structural configurations of CPB under different electronic states. Experimental vibronic spectra were recorded using the (1 + 1) REMPI technique. Additionally, the cation spectra of CPB in its D_0_ ground state were acquired using the (1 + 1′) photoelectron velocity-map imaging (VMI) technique, offering a comprehensive view of the molecular properties in this state. Notably, the spectral analysis revealed the exclusive presence of the bisected conformer in both experimental and theoretical data, reinforcing the consistency between the observed spectra and computational predictions. Furthermore, the vibrational features present in the S_1_ and D_0_ states were effectively identified and assigned, enhancing our understanding of the vibrational dynamics within CPB across different electronic states. The precise determination of transition energies associated with the S_0_ → S_1_ and S_1_ → D_0_ transitions further enriched the spectral characterization, providing crucial information on the electronic transitions occurring within the CPB molecule.

## 2. Materials and Methods

The experimental setup employed in this work is based on a custom-built photoelectron velocity-map imaging and time-of-flight mass spectrometer, as detailed in our previous work [14,15,16]. The methodology closely adhered to the procedures outlined in our earlier investigations. CPB (99%) was obtained from Sigma-Aldrich (Shanghai, China). The sample was introduced into a mixture of argon and helium carrier gas environment maintained at a back pressure of approximately 3 atm. To initiate the experiment, a supersonic molecular beam of CPB was generated through a pulsed Parker valve. Subsequently, a skimmer with a diameter of 0.5 mm was employed to precisely collimate the molecular beam before entering the VMI optics assembly. Within the laser interaction region, CPB underwent ionization by the absorption of two ultraviolet (UV) photons, a crucial step in the experimental process.

Two tunable UV lasers were employed in the present experiments. The excitation laser was generated through frequency doubling the output of a tunable dye laser (Sirah, Grevenbroich, Germany). This dye laser was optically pumped by a Nd: YAG laser. A KDP crystal was used to double the frequency of the visible radiation, producing the UV beam, which was then introduced unfocused into the ionization zoom of the molecular beam. The ionization laser was produced by frequency doubling of a second dye laser, pumped by the 532 nm fundamental of another Nd: YAG laser. The visible wavelengths were calibrated by a wavemeter (HighFinesse, WS 5, Tübingen, Germany), which provides an absolute accuracy of better than ±0.2 cm^−1^ in the fundamental. After frequency doubling, the absolute uncertainty in the UV wavelength was estimated to be approximately ±0.4 cm^−1^. During the experiments, the spot size of the laser beam was adjusted to approximately 1 mm in diameter. The laser had a bandwidth of about 0.1 cm^−1^ and a pulse duration of about 6 ns, and it was operated at a repetition rate of 10 Hz.

During the (1 + 1) REMPI experiments, CPB was excited to a specific resonant vibronic level in the S_1_ state by absorbing a UV photon, after which a second photon of identical wavelength ionized the molecule. The resulting signals from the CPB cations were measured by a microchannel plate detector. Vibronic spectra were acquired by scanning the laser wavelengths. In the photoelectron VMI experiments [17,18,19,20], the excitation laser excited CPB to the S_1_ origin. Subsequently, the ionization laser was utilized to ionize these excited molecules. To prevent (1 + 1) processes, the energy of the excitation laser was meticulously adjusted to remain below 10 μJ, while the ionization laser was set to non-resonant wavelengths. As a result, photoionization did not occur when only one laser was employed. The photoelectrons generated in this process were subjected to velocity mapping by the VMI optics and subsequently directed onto an imaging detector. The real-time photoelectron images formed on the phosphor screen were recorded using an CCD camera. These photoelectron signals underwent real-time ion-counting processing similar to established procedures [21] and were accumulated to create a complete raw image. The cation spectra were reconstructed from these photoelectron images using the BASEX program [22]. This reconstruction process allowed for the detailed analysis of the experimental spectra obtained from the VMI experiments.

**Figure 1:**
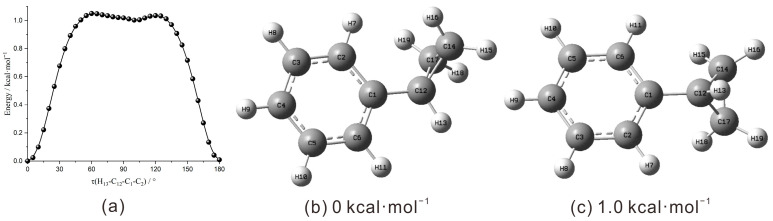
(**a**) Relaxed potential energy surface scan for CPB by varying the dihedral angle τ(H_13_-C_12_-C_1_-C_2_) at the B3LYP-D3(BJ)/def2-TZVP level of theory. Calculated S_0_ neutral ground state structures of (**b**) bisected and (**c**) perpendicular CPB optimized at the B3LYP/aug-cc-pVDZ level of theory.

All quantum chemical computations were performed with the Gaussian 09 software suite [23]. A relaxed potential energy surface scan for CPB was performed by incrementally varying the dihedral angle τ(H_13_-C_12_-C_1_-C_2_) using the B3LYP-D3(BJ)/def2-TZVP level of theory [24,25,26]. The atom numbering scheme for the molecular configurations is illustrated in Figure 1. The potential energy surface scan indicated the existence of two stable conformers. Based on the original geometries, subsequent vibrational analyses and excited-state calculations were performed at a more expensive level of theory. Geometry optimization for the S_0_ and D_0_ states were conducted using DFT methods at the B3LYP/aug-cc-pVDZ level [24,25,27], whereas optimization of the S_1_ excited state was performed by TD-DFT method at the same theoretical level [28,29]. A larger basis set was used to achieve better accuracy in conducting the vibrational and energetic analyses. Vibrational frequencies were derived from the optimized geometries of all three states, and no imaginary frequencies were found. A scaling factor of 0.967 was adopted to the computed harmonic vibrational frequencies based on a previously reported benchmark database [30].

## 3. Results and Discussion

### 3.1. Molecular Structures and Molecular Orbitals

As illustrated in Figure 1a, a relaxed potential energy scan reveals the existence of only two stable conformers for the CPB molecule, the bisecting conformer (τ(H_13_-C_12_-C_1_-C_2_) = 0°, Figure 1b) and the perpendicular conformer (τ(H_13_-C_12_-C_1_-C_2_) = 90°, Figure 1c). The theoretical calculations indicate that the bisected conformer is approximately 1.0 kcal·mol^−1^ more energetically favorable than the perpendicular conformer. According to the systematic investigations of the relaxation of conformers under supersonic jet conditions, an energy difference greater than 1 kcal·mol^−1^ would suppress the existence of the metastable conformer [31]. Unless specified otherwise, “CPB” refers to the bisected conformer in this context. Figure 1b,c present the optimized molecular structures of the bisected and perpendicular conformers of CPB. Across the S_0_, S_1_ and D_0_ states, the bisected conformer maintains *C*_s_ symmetry. The corresponding calculated bond lengths, bond angles, as well as dihedral angles are summarized in Table S4.

**Figure 2:**
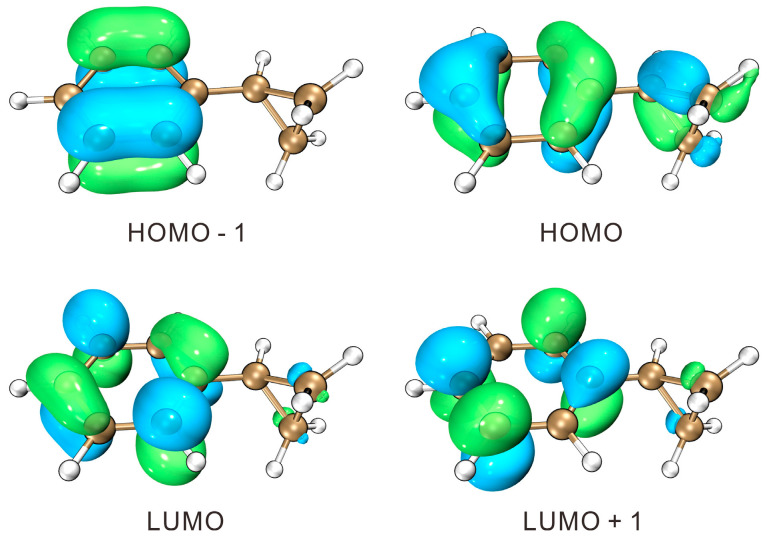
Contour plots of the frontier molecular orbitals of the S_0_ state of CPB (isovalue = 0.05 a.u.). HOMO—highest occupied molecular orbital; LUMO—lowest unoccupied molecular orbital.

The molecular orbital (MO) features of the CPB molecule were analyzed using the Multifunctional Wavefunction Analyzer (Multiwfn, Version 3.8) [32,33] and Visual Molecular Dynamics (VMD, version 1.9.4) [34] software packages. Electronic transition analyses indicate that the S_0_ → S_1_ excitation transition primarily composed of approximately 71% contribution from the HOMO–LUMO transition and 17% from the HOMO − 1–LUMO + 1 transition. Figure 2 presents the contour plots of the principal frontier molecular orbitals that contribute to the S_0_ → S_1_ electronic transition. Both the HOMO and HOMO-1 display apparent π orbital characteristics. The HOMO-1 consists of π electrons from the aromatic ring, while the HOMO is contributed by π electrons from the benzene ring and σ electrons from the cyclopropyl ring. The maximum overlap of the σ electrons and π electrons serves to stabilize the molecule. Electron donation from the three-membered ring results in the elongation of the vicinal C_12_–C_14_ and C_12_–C_17_ bonds compared to the distal C_14_–C_17_ bond. The LUMO and LUMO + 1 exhibit significant π* character, originating from the p orbitals of the benzene ring carbons. As is typical for benzene derivatives, the π → π* excitation induced expansions of the benzene ring, resulting in the elongation of all C–C bonds within the benzene ring. Compared with the S_0_ geometry, all the C–C bonds of the benzene ring in the S_1_ state are lengthened. Upon the S_1_ → D_0_ transition, the elimination of an electron extends the neighboring C_12_–C_14_ and C_12_–C_17_ bonds while contracts the distant C_14_–C_17_ bond. Throughout excitation and ionization, there are no significant alterations in bond angles or dihedral angles.

### 3.2. Vibrational Spectrum of CPB in the S_1_ State

**Figure 3:**
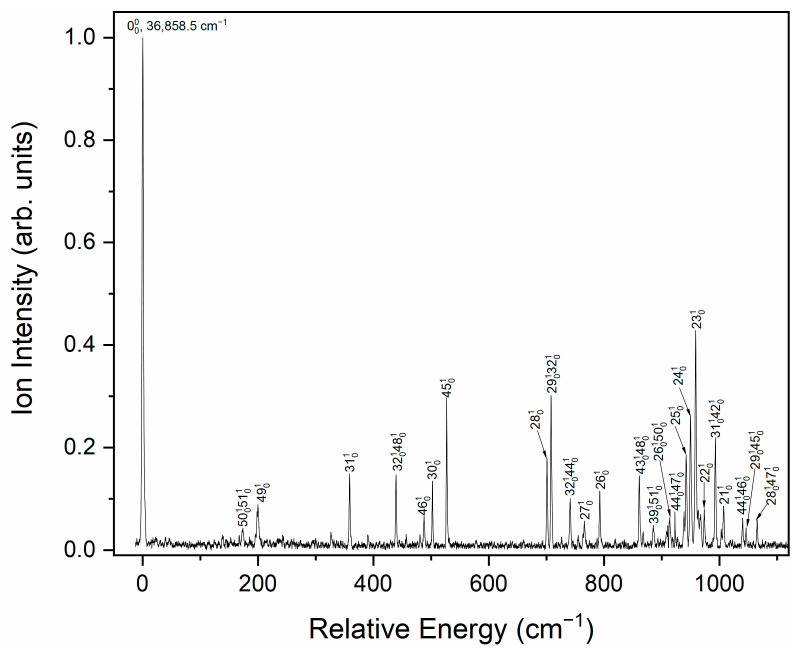
Vibronic spectrum of jet-cooled CPB. The band origin of the S_0_ → S_1_ transition appears at 36,858.5 cm^−1^.

The vibrationally resolved electronic spectra of CPB were recorded in the 263.0–271.4 nm region using the (1 + 1) REMPI technique, as illustrated in Figure 3. These spectra resemble previously reported REMPI spectra measured by Philis et al. [8] and Villani et al. [9], although some weak vibrational structures were also observed in our spectra. The most prominent peak at 36,858.5 cm^−1^ is recognized as the band origin corresponding to the S_0_ → S_1_ electronic transition, aligning with the previously reported value of 36,860 cm^−1^ [8,9]. Notably, only the most stable bisected conformer was detected under the expansion jet conditions, and no evidence supports the existence of the metastable perpendicular conformer. We have performed a conformation weight analysis based on the relative energies calculated at the B3LYP/aug-cc-pVDZ level using the Shermo software (Version 2.4) [35]. The energy difference (~1 kcal/mol) suggests that the bisected conformer is the predominant species in the ground state (>99%), and the perpendicular conformer can be ignored, because its Boltzmann weight is close to zero due to its high energy. The potential energy scan reveals that the barrier to interconversion between the bisected and perpendicular conformers is not higher than 1 kcal·mol^−1^, the higher energy conformer may relax to the most stable bisected form under supersonic jet conditions [31]. By comparing the experimental data with previous REMPI spectra and theoretical calculations, the vibronic bands of the S_1_ electronic excited state of CPB were assigned. Table 1 summarizes these results, while Figure S2 and Table S2 provide detailed information on the calculated vibrational structures and frequencies for the bisected conformer of CPB. The Mulliken convention was adopted for the numbering of vibrational modes in the present work.

The bands observed at 199.6, 487.7, and 526.7 cm^−1^ correspond to out-of-plane fundamental vibrational modes 49^1^, 46^1^, and 45^1^, respectively. Mode 49^1^ is associated with the torsion of the cyclopropyl ring and the bending motions of C–H bonds in the benzene ring. Modes 46^1^ and 45^1^ arise from C–H bending vibrations within the benzene ring. The peaks at 358.8, 502.3, 700.9, 765.5, 792.5, 942.3, 949.8, 958.7, 973.5, and 1007.2 cm^−1^ are attributed to in-plane fundamental vibrational modes 31^1^, 30^1^, 28^1^, 27^1^, 26^1^, 25^1^, 24^1^, 23^1^, 22^1^, and 21^1^, respectively. Mode 27^1^ primarily corresponds to C–H bending motions in the cyclopropyl ring, while the other modes originate from C–H bending motions in both the cyclopropyl and benzene rings. The remaining bands are attributed to combination vibrational modes, as illustrated in Figure 3 and detailed in Table 1.

### 3.3. Photoelectron Spectra via the S_0_ → S_1_ Origin

**Figure 4:**
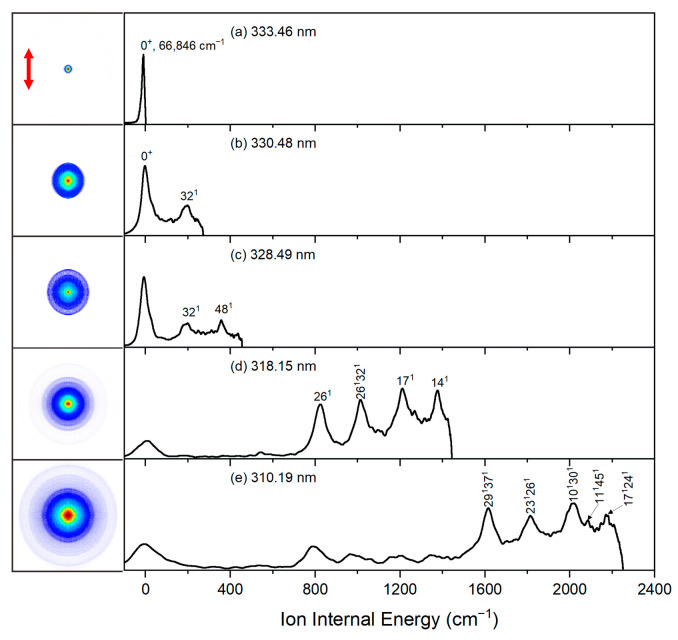
Photoelectron images and corresponding cation spectra of CPB via the S_1_ 0^0^ intermediate state, captured with ionization laser wavelengths at (**a**) 333.46 nm, (**b**) 330.48, (**c**) 328.49 nm, (**d**) 318.15 nm, and (**e**) 310.19 nm. The red double arrow shows the polarization directions of the excitation and ionization lasers.

**Figure 5:**
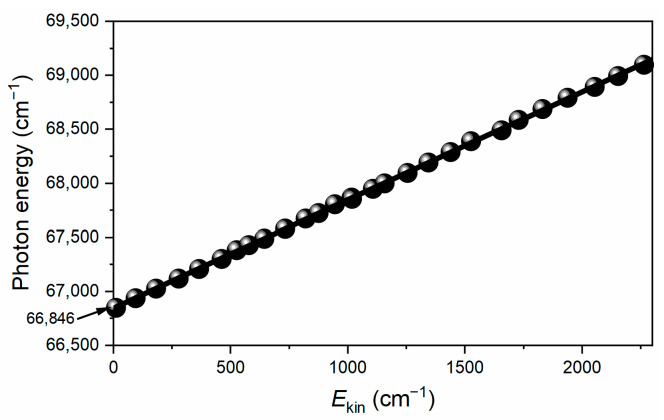
Linear regression analysis between the total photon energy and photoelectron kinetic energy of the ground state cation yields the adiabatic ionization energy to be 66,846 ± 15 cm^−1^.

When exciting and ionizing CPB molecules, the conservation of energy can be expressed by the equation: *hν*_pump_ + *hν*_probe_ − AIE = *E*_int_ + *E*_kin_, where *hν*_pump_ represents the excitation photon energy, *hν*_probe_ the ionization photon energy, AIE the adiabatic ionization energy, *E*_int_ the internal energy of the resulting CPB cations, and *E*_kin_ the photoelectron kinetic energy. Consequently, photoelectron images exhibit discernible rings that correspond to specific vibrational energy levels of the CPB cations. As a result, cation spectra are obtained by recording photoelectron images at various laser wavelengths. Notably, the outermost ring specifically corresponds to the vibrationally ground state of the CPB cations (ν = 0, *E*_kin_ = 0). To determine the AIE value of CPB, multiple photoelectron images were obtained at different photoionization wavelengths via S_0_ → S_1_ excitation. Figure 4 displays the original photoelectron images and photoelectron spectra of CPB ionizing at (a) 333.46 nm, (b) 330.48, (c) 328.49 nm, (d) 318.15 nm, and (e) 310.19 nm. Additionally. Figure 5 depicts the correlation between the total photon energy (*hν*_pump_ + *hν*_probe_) and the photoelectron kinetic energy (*E*_kin_) of the outermost rings. Through linear regression analysis, the AIE value of CPB were yielded as 66,846 ± 15 cm^−1^ (8.288 ± 0.002 eV), which is approximately 0.01 eV lower than that measured by electron impact ionization [36].

Figure 4 presents the photoelectron images captured at five selected photoionization wavelengths, while exciting at the S_1_ 0^0^ band origin. From these images, the corresponding cation spectra were reconstructed using the inverse Abel transform method. Vibrational assignments for the cation spectra were conducted by analyzing experimental data and theoretical vibrational frequencies. The assignments were summarized in Table 2 (All vibrational modes of bisected CPB conformer in the S_0_, S_1_, and D_0_ states are detailed in Figures S1–S3 and Tables S1–S3). The peaks at 201, 360, 564, 823, 1210, and 1377 cm^−1^ were assigned to fundamental modes 32^1^, 48^1^, 29^1^, 26^1^, 17^1^, and 14^1^, respectively. Vibrational mode 32^1^ arises from in-plane C–H and C–C bending vibrations, exhibiting the wagging of benzene and cyclopropyl rings. Mode 48^1^ is attributed to out-of-plane C–H bending vibration, causing the benzene ring deformation. Mode 29^1^ arise from in-plane C–H bending vibration and represents the benzene ring breathing mode. Mode 26^1^ is associated with in-plane C–C stretching and C–H bending vibrations, causing the benzene and cyclopropyl ring breathing. Mode 17^1^ corresponds to in-plane C–H bending vibration. Mode 14^1^ is due to in-plane C–C stretching and C–H bending vibration. Other well-resolved spectral features at 1013, 1615, 1809, 2016, 2083, and 2179 cm^−1^ were assigned to combination vibrational modes.

## 4. Conclusions

In conclusion, the vibronic and cation spectra of gas-phase jet-cooled cyclopropylbenzene (CPB) were successfully recorded using REMPI spectroscopy and photoelectron VMI spectroscopy techniques in combination with theoretical calculations. The experimental results confirmed the presence of only the most stable bisected conformer of CPB under the specific experimental conditions of the study. The band origin corresponding to the S_0_ → S_1_ electronic transition was observed at 36,858.5 cm^−1^. Moreover, the adiabatic ionization energy was measured to be 66,846 ± 15 cm^−1^, providing valuable insights into the photoionization properties of the molecule. Furthermore, the active vibrational modes of CPB in the S_1_ and D_0_ states were successfully assigned through a comprehensive analysis combining experimental data with theoretical calculations. These detailed vibrational assignments enhanced our understanding of the molecular structure and dynamics of CPB, shedding light on its behavior in different electronic states.

## Figures and Tables

**Table 1:** Observed vibronic bands and assignments of the REMPI spectrum of CPB.

Energy (cm^−1^)	Shift	ω (Calc.) ^1^	Assignment	Ref. [9]	Ref. [8]
36,858.5	0		0^0^, origin		
37,032.2	173.7	169	50^1^51^1^		
37,058.1	199.6	194	49^1^	200	199
37,217.3	358.8	356	31^1^	358	358
37,297.6	439.1	447	32^1^48^1^	440	439
37,346.2	487.7	486	46^1^		
37,360.8	502.3	500	30^1^	502	500
37,385.4	526.9	532	45^1^	527	527
37,559.4	700.9	695	28^1^		701
37,566.4	707.9	705	29^1^32^1^	708	706
37,599.6	741.1	743	32^1^44^1^		
37,624.0	765.5	771	27^1^		
37,651.0	792.5	796	26^1^		
37,719.5	861.0	858	43^1^48^1^	860	860
37,743.9	885.4	884	39^1^51^1^		
37,772.2	913.7	912	26^1^50^1^		
37,781.1	922.6	924	44^1^47^1^		
37,800.8	942.3	942	25^1^		942
37,808.3	949.8	953	24^1^		950
37,817.2	958.7	963	23^1^	958	958
37,826.0	967.5	965	27^1^49^1^		
37,832.0	973.5	970	22^1^		
37,851.4	992.9	991	31^1^42^1^		993
37,861.6	1003.1	1001	29^1^46^1^		
37,865.7	1007.2	1004	21^1^		
37,898.5	1040.0	1039	44^1^46^1^		
37,904.6	1046.1	1047	29^1^45^1^		
37,923.7	1065.2	1066	28^1^47^1^		

^1^ Calculated at the B3LYP/aug-cc-pVDZ level of theory, scaled by 0.967.

**Table 2:** Vibrational assignments of the D_0_ state of CPB observed in the photoelectron spectra.

Shift	ω (Calc.) ^1^	Assignment ^2^
0		0^0^, origin
201	188	32^1^, *β*(CH), *β*(CC), ring wagging
360	358	48^1^, *γ*(CH)
564	559	29^1^, *β*(CH), benzene ring breathing
823	824	26^1^, *β*(CH), *ν*(CC), benzene and cyclopropyl ring breathing
1013	1012	26^1^32^1^
1210	1211	17^1^, *β*(CH)
1377	1377	14^1^, *β*(CH), *ν*(CC)
1615	1609	29^1^37^1^
1809	1804	23^1^26^1^
2016	2012	10^1^30^1^
2083	2083	11^1^45^1^
2179	2177	17^1^24^1^

^1^ Theoretical vibrational frequencies were obtained from the B3LYP/aug-cc-pVDZ calculations, scaled by 0.967. All units are in cm^−1^. ^2^ ν, β, and γ indicate stretching, in-plane bending, and out-of-plane bending vibrations, respectively.

## Data Availability

Data underlying the results presented in this paper are not publicly available at this time but may be obtained from the authors upon reasonable request.

## Supplementary Materials

The following supporting information can be downloaded at: https://www.mdpi.com/article/10.3390/molecules31101658/s1, Figure S1: The displacement vectors of the vibrational normal modes of bisected CPB in the S_0_ neutral ground state and their calculated harmonic frequencies (scaled by 0.967); Figure S2: The displacement vectors of the vibrational normal modes of bisected CPB in the S_1_ first excited state and their calculated harmonic frequencies (scaled by 0.967); Figure S3: The displacement vectors of the vibrational normal modes of bisected CPB in the D_0_ cationic ground state and their calculated harmonic frequencies (scaled by 0.967); Table S1: Theoretical vibrational frequencies for the S_0_ ground state of bisected CPB calculated at the DFT/B3LYP/aug-cc-pVDZ level of theory with a scaling factor of 0.967; Table S2: Theoretical vibrational frequencies for the S_1_ excited state of bisected CPB calculated at the TD-DFT/B3LYP/aug-cc-pVDZ level of theory with a scaling factor of 0.967; Table S3: Theoretical vibrational frequencies for the D_0_ cationic ground state of bisected CPB calculated at the DFT/B3LYP/aug-cc-pVDZ level of theory with a scaling factor of 0.967. Table S4: Calculated geometrical parameters of bisected CPB conformer in the S_0_, S_1_, and D_0_ states.
