# Supplementary material for: Vibronic and Cation Spectra of Cyclopropylbenzene Conformer
Source: Molecules. 2026 May 14;31(10):1658. doi: 10.3390/molecules31101658 (PMC13209889; doi:10.3390/molecules31101658)
Supplement: Supplementary file 1 [file molecules-31-01658-s001.zip › molecules-4290173-supplementary.pdf]

Supporting Information for:

Vibronic and cation spectra of cyclopropylbenzene conformer

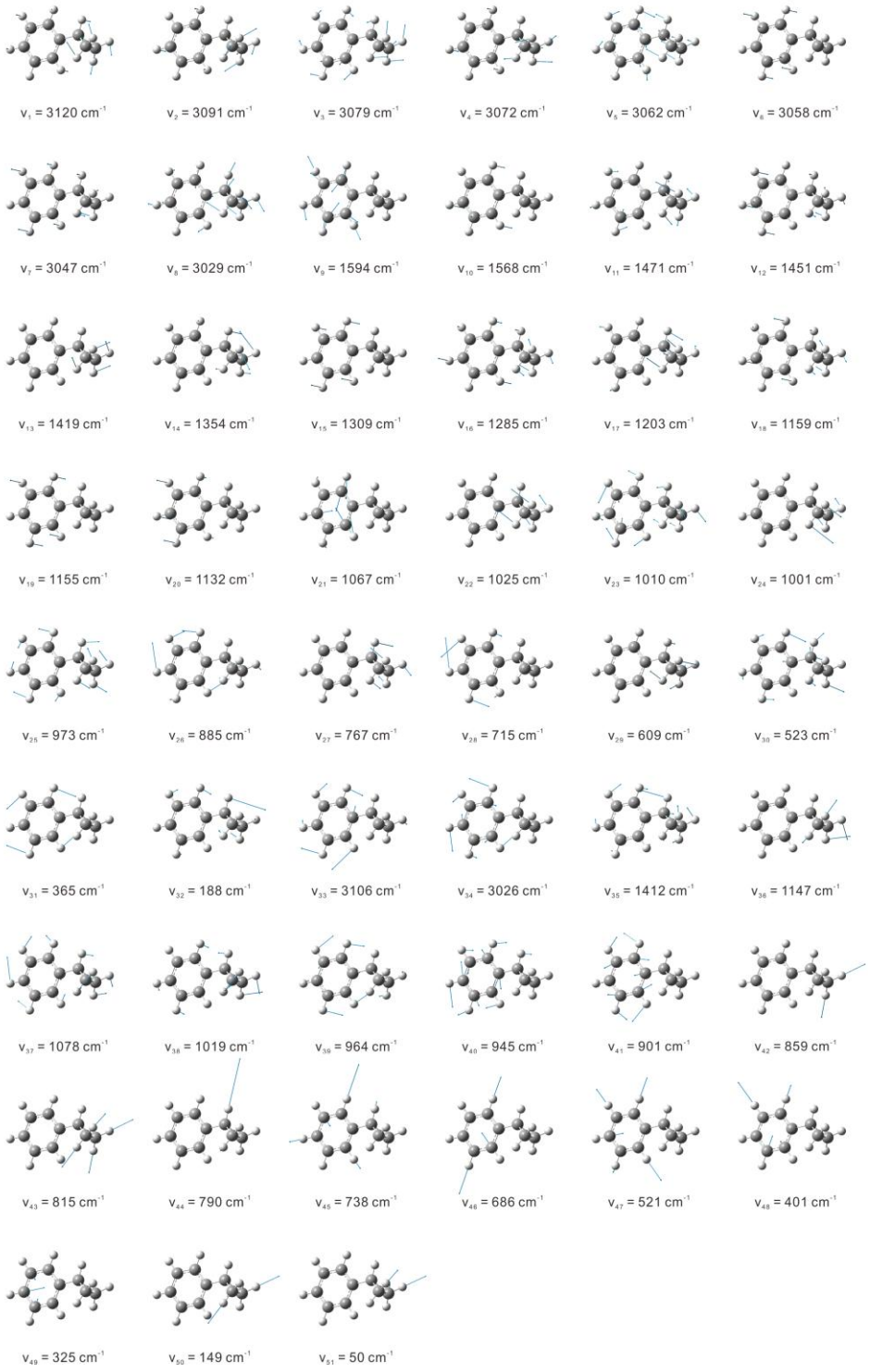

Figure S1. The displacement vectors of the vibrational normal modes of bisected CPB in the S_0 neutral ground state and their calculated harmonic frequencies (scaled by 0.967).

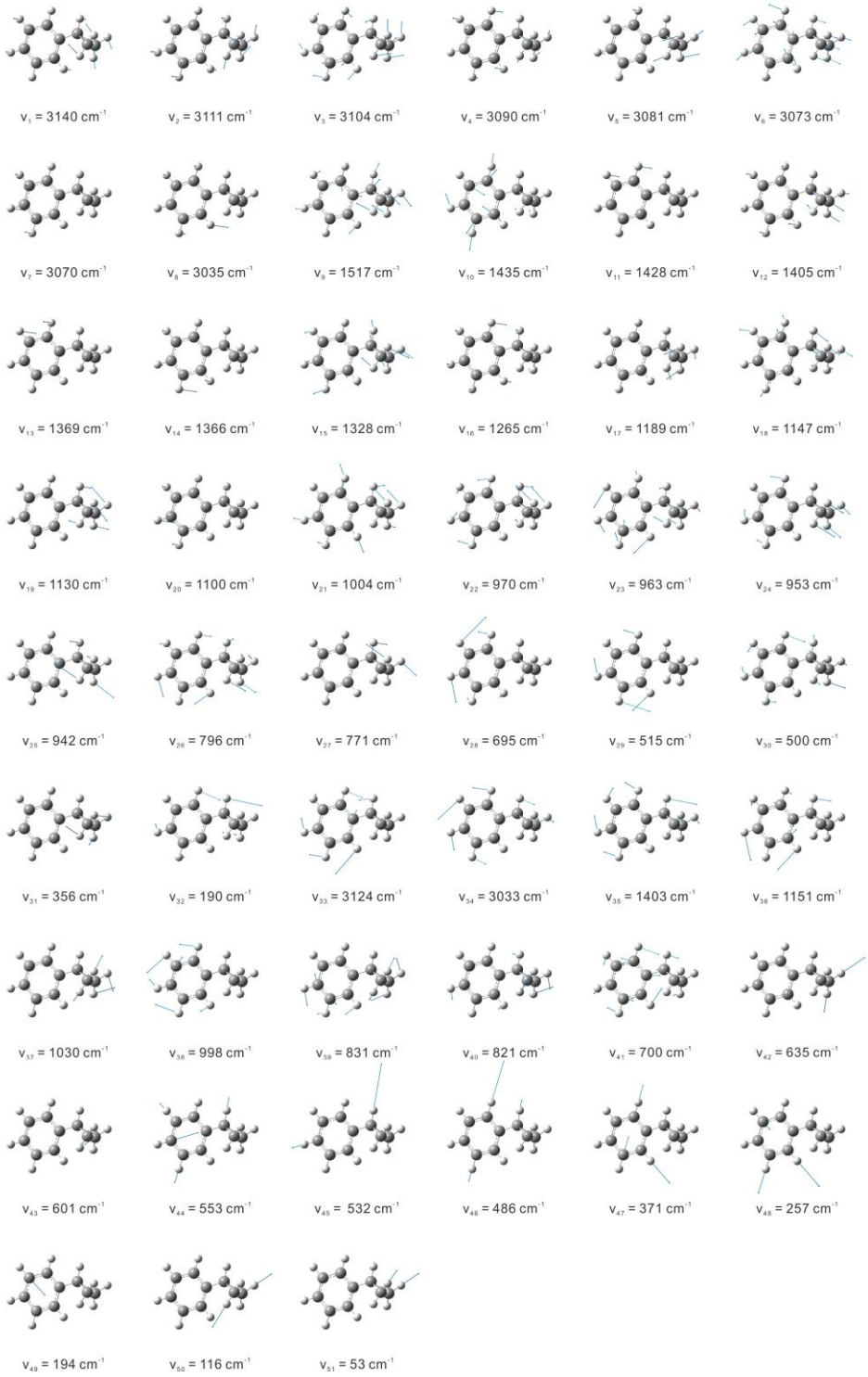

Figure S2. The displacement vectors of the vibrational normal modes of bisected CPB in the S_1 first excited state and their calculated harmonic frequencies (scaled by 0.967).

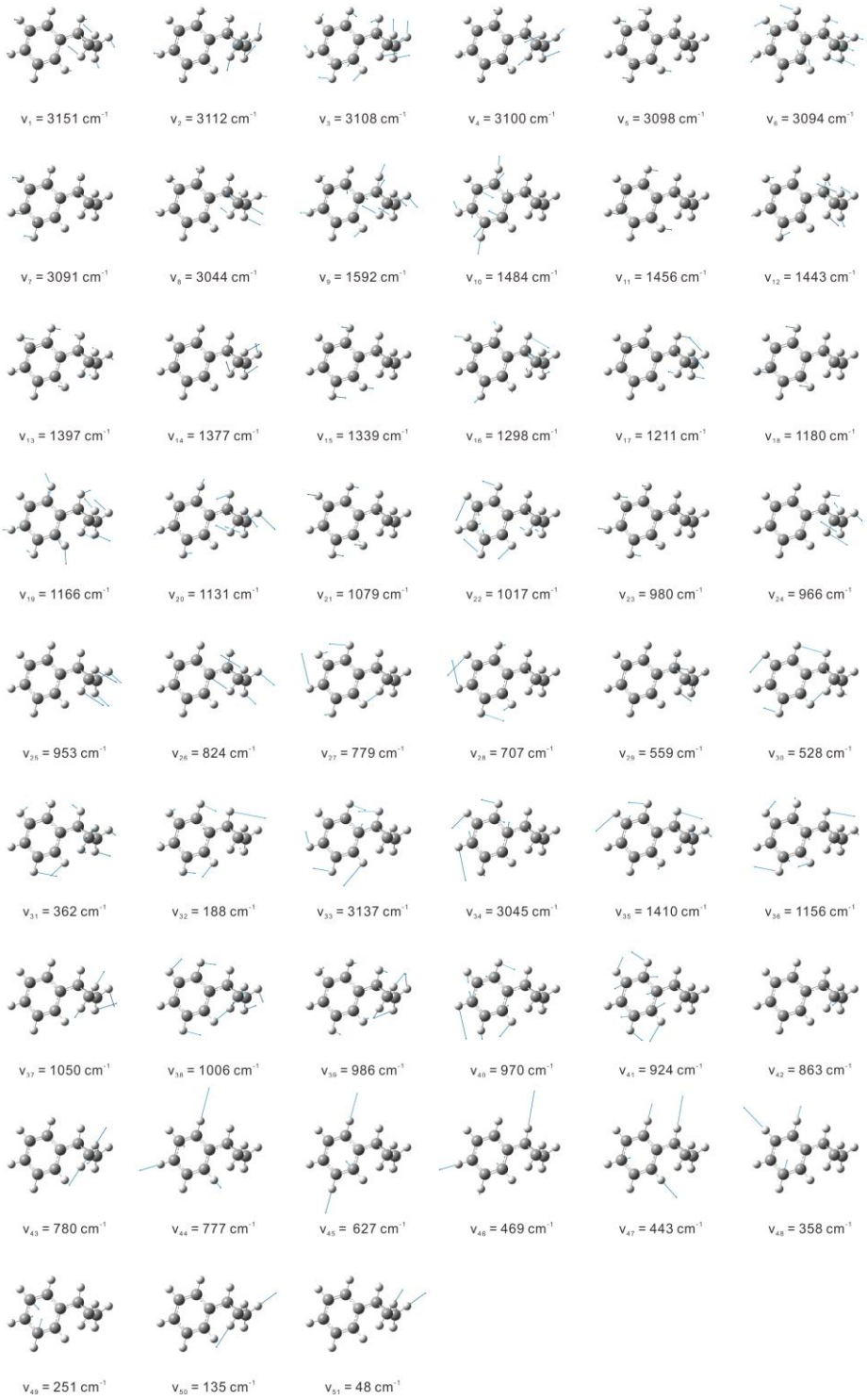

Figure S3. The displacement vectors of the vibrational normal modes of bisected CPB in the D_0 cationic ground state and their calculated harmonic frequencies (scaled by 0.967).

Table S1. Theoretical vibrational frequencies for the S_0 ground state of bisected CPB calculated at the DFT/B3LYP/aug-cc-pVDZ level of theory with a scaling factor of 0.967.

Mode	Symmetry	Frequency (cm^{-1})	Mode	Symmetry	Frequency (cm^{-1})
ν_1	a′	3120	ν_{33}	a″	3106
ν_2		3091	ν_{34}		3026
ν_3		3079	ν_{35}		1412
ν_4		3072	ν_{36}		1147
ν_5		3062	ν_{37}		1078
ν_6		3058	ν_{38}		1019
ν_7		3047	ν_{39}		964
ν_8		3029	ν_{40}		945
ν_9		1594	ν_{41}		901
ν_{10}		1568	ν_{42}		859
ν_{11}		1471	ν_{43}		815
ν_{12}		1451	ν_{44}		790
ν_{13}		1419	ν_{45}		738
ν_{14}		1354	ν_{46}		686
ν_{15}		1309	ν_{47}		521
ν_{16}		1285	ν_{48}		401
ν_{17}		1203	ν_{49}		325
ν_{18}		1159	ν_{50}		149
ν_{19}		1155	ν_{51}		50
ν_{20}		1132			
ν_{21}		1067			
ν_{22}		1025			
ν_{23}		1010			
ν_{24}		1001			
ν_{25}		973			
ν_{26}		885			
ν_{27}		767			
ν_{28}		715			
ν_{29}		609			
ν_{30}		523			
ν_{31}		365			
ν_{32}		188			

Table S2. Theoretical vibrational frequencies for the S_1 excited state of bisected CPB calculated at the TD-DFT/B3LYP/aug-cc-pVDZ level of theory with a scaling factor of 0.967.

Mode	Symmetry	Frequency (cm^{-1})	Mode	Symmetry	Frequency (cm^{-1})
ν_1	a′	3140	ν_{33}	a″	3124
ν_2		3111	ν_{34}		3033
ν_3		3104	ν_{35}		1403
ν_4		3090	ν_{36}		1151
ν_5		3081	ν_{37}		1030
ν_6		3073	ν_{38}		998
ν_7		3070	ν_{39}		831
ν_8		3035	ν_{40}		821
ν_9		1517	ν_{41}		700
ν_{10}		1435	ν_{42}		635
ν_{11}		1428	ν_{43}		601
ν_{12}		1405	ν_{44}		553
ν_{13}		1369	ν_{45}		532
ν_{14}		1366	ν_{46}		486
ν_{15}		1328	ν_{47}		371
ν_{16}		1265	ν_{48}		257
ν_{17}		1189	ν_{49}		194
ν_{18}		1147	ν_{50}		116
ν_{19}		1130	ν_{51}		53
ν_{20}		1100			
ν_{21}		1004			
ν_{22}		970			
ν_{23}		963			
ν_{24}		953			
ν_{25}		942			
ν_{26}		796			
ν_{27}		771			
ν_{28}		695			
ν_{29}		515			
ν_{30}		500			
ν_{31}		356			
ν_{32}		190			

Table S3. Theoretical vibrational frequencies for the D_0 cationic ground state of bisected CPB calculated at the DFT/B3LYP/aug-cc-pVDZ level of theory with a scaling factor of 0.967.

Mode	Symmetry	Frequency (cm^{-1})	Mode	Symmetry	Frequency (cm^{-1})
ν_1	a′	3151	ν_{33}	a″	3137
ν_2		3112	ν_{34}		3045
ν_3		3108	ν_{35}		1410
ν_4		3100	ν_{36}		1156
ν_5		3098	ν_{37}		1050
ν_6		3094	ν_{38}		1006
ν_7		3091	ν_{39}		986
ν_8		3044	ν_{40}		970
ν_9		1592	ν_{41}		924
ν_{10}		1484	ν_{42}		863
ν_{11}		1456	ν_{43}		780
ν_{12}		1443	ν_{44}		777
ν_{13}		1397	ν_{45}		627
ν_{14}		1377	ν_{46}		469
ν_{15}		1339	ν_{47}		443
ν_{16}		1298	ν_{48}		358
ν_{17}		1211	ν_{49}		251
ν_{18}		1180	ν_{50}		135
ν_{19}		1166	ν_{51}		48
ν_{20}		1131			
ν_{21}		1079			
ν_{22}		1017			
ν_{23}		980			
ν_{24}		966			
ν_{25}		953			
ν_{26}		824			
ν_{27}		779			
ν_{28}		707			
ν_{29}		559			
ν_{30}		528			
ν_{31}		362			
ν_{32}		188			

Table S4. Calculated geometrical parameters of bisected CPB conformer in the S_0, S_1, and D_0 states.

	S_0	S_1	D_0
**Bond length (Å)**			
C_1–C_2	1.407	1.438	1.438
C_2–C_3	1.397	1.429	1.377
C_3–C_4	1.399	1.417	1.417
C_4–C_5	1.397	1.419	1.414
C_5–C_6	1.398	1.430	1.378
C_1–C_6	1.405	1.430	1.438
C_1–C_{12}	1.492	1.453	1.431
C_{12}–C_{14}	1.520	1.563	1.594
C_{12}–C_{17}	1.520	1.563	1.594
C_{14}–C_{17}	1.505	1.476	1.451
C_2–H_7	1.091	1.088	1.089
C_3–H_8	1.091	1.089	1.089
C_4–H_9	1.090	1.091	1.090
C_5–H_{10}	1.091	1.088	1.089
C_6–H_{11}	1.091	1.090	1.090
C_{12}–H_{13}	1.090	1.089	1.088
C_{14}–H_{15}	1.089	1.090	1.090
C_{14}–H_{16}	1.089	1.088	1.088
C_{17}–H_{18}	1.089	1.090	1.090
C_{17}–H_{19}	1.089	1.088	1.088
**Bond angle (°)**			
C_2–C_1–C_6	117.9	120.2	118.5
C_1–C_2–C_3	121.0	119.0	120.4
C_2–C_3–C_4	120.4	119.9	120.0
C_3–C_4–C_5	119.2	121.8	120.7
C_4–C_5–C_6	120.2	118.6	119.7
C_1–C_6–C_5	121.2	120.4	120.7
C_2–C_1–C_{12}	122.6	121.9	122.9
C_6–C_1–C_{12}	119.5	117.9	118.6
C_1–C_{12}–C_{14}	121.8	122.0	121.5
C_1–C_{12}–C_{17}	121.8	122.0	121.5
C_{14}–C_{12}–C_{17}	59.4	56.4	54.2
C_{12}–C_{14}–C_{17}	60.3	61.8	62.9
C_{12}–C_{17}–C_{14}	60.3	61.8	62.9
**Dihedral angle (°)**			
H_{13}–C_{12}–C_1–C_2	180.0	180.0	180.0
H_{13}–C_{12}–C_1–C_6	0.0	0.0	0.0
